# Patient and family engagement interventions in primary care patient safety: systematic review and meta-analysis of randomised controlled trials

**DOI:** 10.3399/BJGP.2024.0369

**Published:** 2025-02-18

**Authors:** Yan Pang, Anna Szücs, Ignacio Ricci-Cabello, Jaheeda Gangannagaripalli, Lay Hoon Goh, Foon Leng Leong, Li Fan Zhou, Jose Maria Valderas

**Affiliations:** Department of Family Medicine, National University Health System (NUHS), Singapore, Singapore; Alice Lee Centre for Nursing Studies, Yong Loo Lin School of Medicine, National University of Singapore, Kent Ridge, Singapore; Department of Family Medicine, National University Health System (NUHS), Singapore, Singapore; Division of Family Medicine, Department of Medicine, Yong Loo Lin School of Medicine, National University of Singapore, Kent Ridge, Singapore; Faculty of Behavioural and Movement Sciences, Vrije Universiteit Amsterdam, Amsterdam, the Netherlands; Health Research Institute of the Balearic Islands (IdISBa), Balearic Islands, Spain; Balearic Islands Cochrane Affiliated Centre, Palma, Spain; CIBER de Epidemiología y Salud Pública (CIBERESP), Institute of Health Carlos III, Madrid, Spain; NIHR Applied Research Collaboration Greater Manchester (ARC-GM), Manchester, UK; Healthy Ageing Research Group, School of Health Sciences, Faculty of Biology, Medicine and Health, University of Manchester, Manchester, UK; Manchester Academic Health Science Centre, Manchester, UK; Department of Family Medicine, National University Health System (NUHS), Singapore, Singapore; Division of Family Medicine, Department of Medicine, Yong Loo Lin School of Medicine, National University of Singapore, Kent Ridge, Singapore; National University Polyclinics (NUP), Singapore, Singapore; Department of Family Medicine, National University Health System (NUHS), Singapore, Singapore; Division of Family Medicine, Department of Medicine, Yong Loo Lin School of Medicine, National University of Singapore, Kent Ridge, Singapore; Department of Statistics & Data Science, National University of Singapore, Kent Ridge, Singapore; Department of Family Medicine, National University Health System (NUHS), Singapore, Singapore; Division of Family Medicine, Department of Medicine, Yong Loo Lin School of Medicine, National University of Singapore, Kent Ridge, Singapore; Centre for Research in Health Systems Performance (CRiHSP), National University of Singapore, Singapore, Singapore

**Keywords:** family engagement, family involvement, family medicine, patient engagement, patient involvement, patient safety, randomised controlled trial, patient participation, primary health care

## Abstract

**Background:**

Engaging patients and families has been promoted as a key strategy for improving patient safety of health systems. However, evidence remains scarce on the effectiveness of this approach in primary care.

**Aim:**

To assess the combined effectiveness of primary care interventions in randomised controlled trials (RCTs) promoting patient and family engagement in patient safety.

**Design and setting:**

A systematic review and meta-analysis.

**Method:**

The review followed PRISMA and Cochrane guidelines. Five electronic databases (Medline, CINAHL, Embase, Web of Science, CENTRAL) were searched from inception to 18 September 2024 with keywords in four blocks (patient and family engagement; patient safety; primary care; randomised controlled trial). Patient and family engagement levels were appraised. Where appropriate, results were combined into meta-analyses.

**Results:**

Of the 19 included records, 12 reported on completed RCTs. Only one intervention integrated patients/families into overall care safety (high engagement); six aimed at enhancing skills and tools (intermediate), and 12 informed patients/families how to engage and prompted them to do it (low). RCTs primarily targeted medication safety, with meta-analyses showing no significant effects on reducing adverse drug events (odds ratio [OR] 0.86, 95% confidence interval [CI] = 0.70 to 1.08) or improving medication appropriateness measured categorically (OR 0.92, 95% CI = 0.76 to 1.13) or continuously (mean difference 0.71, 95% CI = −0.10 to 1.52). Overall risk of bias was low and certainty of evidence very low to moderate.

**Conclusion:**

Existing randomised controlled evidence on patient and family engagement in primary care remains inconclusive and limited in scope. Future interventions should include higher levels of engagement and address more diverse patient safety outcomes relevant for primary care.

## Introduction

Patient safety has gained momentum in past decades, with patient safety strategies being integrated into the agendas of healthcare organisations worldwide.^1^ Yet, the development of strategies and interventions to improve patient safety in healthcare delivery has by and large been confined to hospital care,^2^,^3^ despite 2–3% of primary care consultations being associated with a patient safety incidentwith <1% to 44% resulting in severe harm.^4^ Compared with hospital care, primary care presents unique challenges for patient safety as patient encounters or consultations are usually short, spaced out, sometimes occurring remotely, and usually involving a single provider at each given time, which can precipitate unnoticed prescription or documentation errors, communication gaps, and failures to appraise clinical situations adequately.^5^,^6^

There is a growing recognition of the potential contribution of patients to care and care safety,^7^ which may be particularly relevant to primary and community care.^8^,^9^ The delivery of care within community settings relies heavily on the patients and their families,^10^ making them well placed to identify errors or potential risks of harm.

**How this fits in**

‘Engaging Patients for Patient Safety’, the World Health Organization’s theme for the 2023 World Patient Safety Day, likely holds promise to improve primary care patient safety through joint patient–provider efforts, yet how to implement it effectively remains unclear. This systematic review and meta-analysis synthesised evidence from randomised controlled trials investigating patient and family engagement in primary care patient safety. Included studies yielded inconclusive results with no significant combined effect, whereas most reported interventions remained at low levels of patient and family engagement. These results suggest that classic approaches to patient engagement may not be sufficient, with patient–provider collaborations likely needed to devise novel, more patient-centred interventions that could empower patients to contribute to their own safety and that of the whole practice.

Patient and family engagement strategies go beyond raising awareness about care safety and can encompass partnerships between patients/families and healthcare professionals aimed at preventing or mitigating adverse events.^1^ Even though such strategies were already considered a pillar of patient safety in the landmark report of the Institute of Medicine in 1999, ‘To Err is Human’,^11^ only more recently was their importance re-emphasised by the World Health Organization’s Declaration of Astana^12^ and the designation of ‘Engaging patients for patient safety’ as the theme for the 2023 World Patient Safety Day.^13^ According to the Organisation for Economic Co-operation and Development, effective patient involvement could potentially diminish harm by up to 15% in ambulatory care, leading to significant cost savings for the healthcare system.^14^ Primary care is an optimal setting to implement such strategies because of the sustained relationship among care providers, patients, and families that is traditionally at its root.^15^

Specific interventions, such as face-to-face coaching sessions in older adults,^16^ family carer support in dementia,^17^ and the utilisation of eHealth tools for reporting adverse drug effects,^18^ have demonstrated efficacy in engaging patients and families in primary care patient safety, although some authors view the implementation of these strategies as challenging.^2^ However, numerous patient/family engagement strategies such as patient–provider partnerships,^19^,^20^ patient involvement in decision-making,^21^ decision coaching,^22^ patient access to medical records,^23^ and patient-mediated interventions^24^ remain underexplored in primary care, leaving unclear which patient and family engagement interventions are effective for which patient safety outcomes.^25^

The present study aimed to synthesise best evidence regarding patient and family engagement interventions targeting patient safety outcomes in primary care.

## Method

A systematic review was conducted following the PRISMA guidelines. The search protocol was registered on PROSPERO (registration number: CRD42023397495).

Coulter’s definition of patient engagement was used: *‘a set of reciprocal tasks between patients, healthcare professionals, and healthcare organizations working together to promote and support active patient and public involvement in health and healthcare and to strengthen their influence on healthcare decisions, at both the individual and the collective level’*^26^,^27^ and extended it to patients’ families. Patient safety was defined as *‘a health care discipline that aims to prevent and reduce risks, errors and harm that occur to patients during provision of health care’*.^28^

For the current study the authors adapted the engagement framework developed by Kim and colleagues^29^ to appraise the level of engagement of patients/families in each intervention as low, moderate, and high (Box 1). Of the original framework’s five levels, in the current study the lowest one was removed (‘inform [patients] about healthcare’), as it did not involve active patient participation, and the two top ones were merged, (namely, ‘partner – patients become collaborators/consultants in their care’ and ‘integrate – integrates patients and families as full team members in care’), as both reflected patient/family engagement at the level of overall care as opposed to individual care.

Box 1 **Levels of patient and family engagement in patient safety**

**Table:** 

	Levels	Definitions	Level of impact
Level 1	Informing about engagement and prompting for engagement	Patients receiving information (for example, booklets) to learn about their health and increase communication with their care team	Patient’s individual (own) level of care
Level 2	Empowerment	Patients acquiring new skills and/or tools other than information to engage with care team in patient safety	
Level 3	Partnership	Patients involved in patient safety decision-making as collaborators, consultants, or team members	Practice/setting level of care

### Eligibility criteria and study selection

Records of randomised controlled trials (RCTs) and cluster-randomised trials that recruited participants in primary care settings, such as private practices, family medicine clinics, and community/ambulatory care settings associated with general practice, were included. Specialised ambulatory care settings were not eligible.

Besides records reporting on completed trials, published trial protocols, and trial registrations were eligible, as long as a more complete record was not available about the same trial. The most complete available record for each RCT was included and all records that contributed independent participant data (for example, both a pilot RCT and full-scale RCT testing iterations of the same intervention).

Eligible interventions needed to (a) prompt patients and/or families to take actions in the context of their care; (b) focus on patient/family education about engagement (such as, informing about red flags to be signalled to providers); or (c) have patient/family engagement as a component of a complex intervention, as long as it was reported on separately and involved comparable resources to other components. The current study only considered safety-related outcomes: adverse events, such as falls, fractures, admissions to hospital, or death; non-recommended medical practices, such as inappropriate prescriptions; or favourable safety outcomes, such as correction of inappropriate medical practices or errors. The current study relied on the original authors’ definitions of these terms, given their varying definitions in the literature.

Non-English-language studies, review papers, conference abstracts, trials of secondary or tertiary healthcare and specialist outpatient care, interventions exclusively involving healthcare providers or policymakers, and outcomes pertaining to quality of care but not explicitly to patient safety were excluded.

### Data sources and searches

Five electronic databases were searched for potentially eligible studies, including Medline Ovid, CINAHL, EBSCO, Embase Ovid, Web of Science Core Collection, and Cochrane Central Register of Controlled Trials (CENTRAL) in the Cochrane Library. Reference tracking was also perfromed to check for additional eligible records. The search was performed on 7–9 February 2023 and updated on 18 September 2024. The search strategy encompassed four blocks: primary care, patient safety, patient and family engagement, and randomised controlled trial. For ‘primary care’ and ‘patient safety’, key words were adapted from the current authors’ earlier systematic review on primary care patient safety;^30^ for ‘patient and family engagement’, key words were adapted from the team’s ongoing scoping review on patient engagement and from Cochrane reviews on patient involvement,^31^,^32^ which also included key words for families and caregivers; finally, for ‘randomised controlled trial’, a filter devised by Nwosu and colleagues^33^ was employed that reported having a high overall performance for identifying RCTs in Medline (sensitivity 0.86, specificity 0.96),^34^ while further extending it with PubMed’s narrow filter for RCTs (sensitivity 93%, specificity 97%).^35^ Search terms were adapted to include both British and American English spelling. See Supplementary Tables S1 to S5 for the complete search strategy in each database.

### Data extraction and risk of bias

Article screening and data extraction were performed by two independent team members for each article, with discrepancies resolved through consensus meetings. Data extraction followed the Cochrane data collection guidelines^36^ and risk of bias assessment employed the Cochrane Risk of Bias tool version 2, which assesses studies on five domains and an overall judgement domain, and categorises studies into ‘low concern’, ‘some concern’, and ‘high concern’ of bias.^37^ Certainty of evidence was appraised following the GRADE approach.^38^

### Statistical analysis

Records reporting on pilot and full-scale RCTs were grouped by outcomes (continuous versus categorical) and conceptual similarity (inappropriate prescriptions, side effects, others). For groups containing two or more studies, results were combined in meta-analyses using the metafor 4.6–0^39^ and meta 6.2–1^40^ packages in R (version 4.4.1). Following Cochrane guidelines, the longest follow-up time was used. Random-effects models (DerSimonian and Laird) were applied for groups with moderate to substantial heterogeneity (30%≤*I*^2^≤ 75%), and fixed-effects models (Mantel–Haenszel) for groups with no to negligible heterogeneity (*I*^2^≤30%). Groups with considerable heterogeneity (*I*^2^≥75%) were excluded from meta-analysis.^41^,^42^ Categorical outcomes used the natural logarithm of odds ratios (ORs) and corresponding variance to estimate pooled ORs. Meta-analysis of continuous outcomes was reported as mean difference in scores. Records unsuitable for meta-analysis were summarised narratively.

## Results

### Study selection and general characteristics

**Figure 1:**
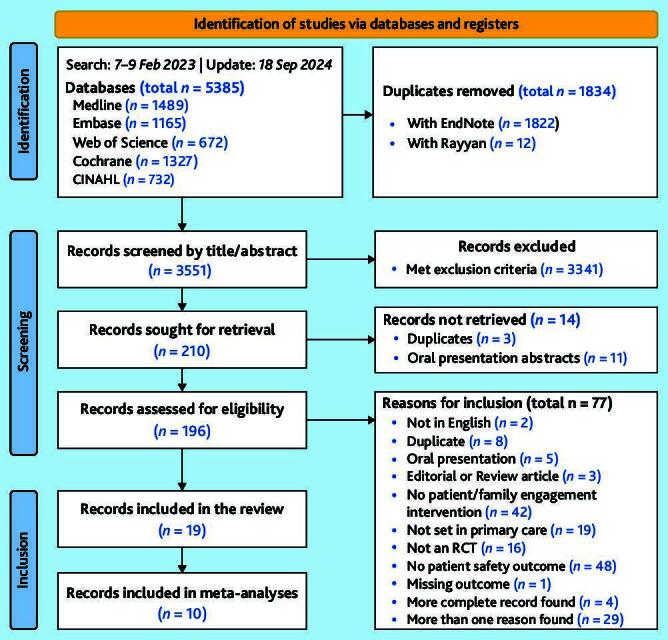
PRISMA Chart summarising the screening process. RCT = randomised controlled trial.

The systematic search yielded a total of 5385 records (4773 from the initial search and 612 from the search update), of which 3551 remained after deduplication and 196 after full-text retrieval (Figure 1). A final set of 19 records were included.^43^–^61^ Raw outcome data on patient safety could not be obtained for one study,^62^ after two unsuccessful attempts to contact the authors by email, leading to this study’s exclusion. Supplementary Table S6 provides a detailed summary of included records.

All included records were published between 2001 and 2024. Twelve records presented completed studies, of which eight were cluster RCTs and four were standard RCTs. Of the remaining seven records, six were RCT protocols, and one was a trial registration.

There were seven records from the US, three from Germany (of which two were from the same research programme), two each from Australia, Switzerland, and Spain, and one each from Canada, France, and the UK. The authors’ country of affiliation matched where projects were carried out for all completed RCTs.

Publications increased with time: two records were published between 2000 and 2014, six between 2015 and 2019, and 11 records between 2020 and 2024.

The follow-up duration across RCTs ranged from 2 weeks to 14 months, and the number of randomised participants from 100 to 11 800. Among 12 completed RCTs, a total of 9391 participants were included, comprising 2120 family units, namely 1599 parent–child dyads^48^ and 521 older participants with family or non-family caregivers.^58^ One study described a joint patient/family intervention in older patients with dementia or mild cognitive impairment, but kept the inclusion of family members optional and did not report on the number of families included.^53^ The combined mean age of participants was 74.2 years (SD = 8.8) based on available data from seven completed RCTs.^44^,^45^,^49^–^51^,^53^,^58^ Mean age was not reported in five completed RCTs, of which three were conducted in adults aged >65 years,^47^,^55^,^56^ one in adults aged >18 years,^57^ and one in children aged between 1 and 5 years.^48^ Among all included interventions, about half (*n* = 9) targeted older patients.^46^,^47^,^49^,^50^,^53^,^55^,^56^,^58^,^61^

### Outcome and intervention characteristics

Patient safety outcomes examined were predominantly adverse drug events (*n* = 10; five completed RCTs) and assessments of medication appropriateness (*n* = 7; all completed RCTs, of which one also reported on adverse drug events.^56^) Other outcomes (*n* = 3; one completed RCT) included medication discrepancy corrections,^55^ avoidable admissions to hospital,^54^,^60^ patient experiences of harm,^54^ as well as care centre-level safety culture and number of initiatives to improve patient safety.^54^

**Figure 2:**
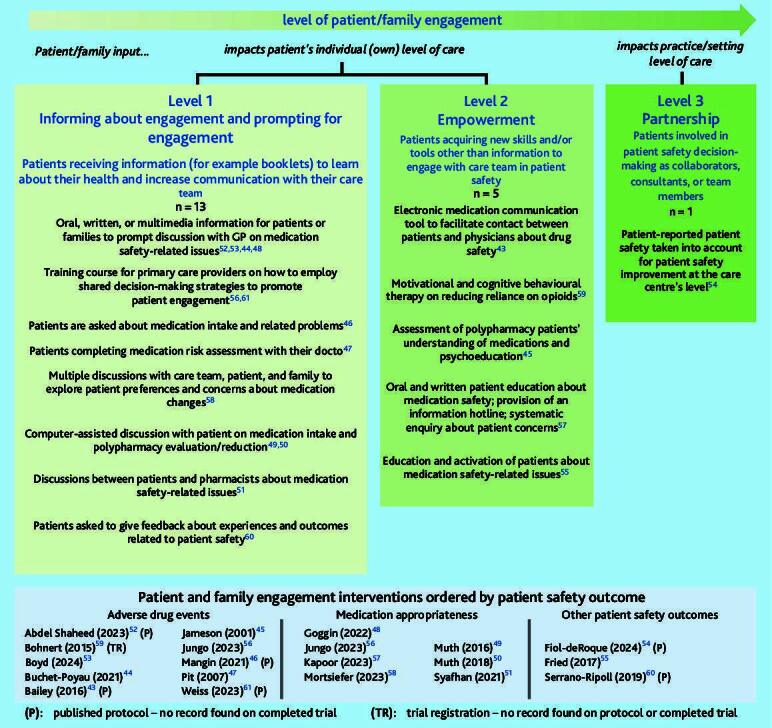
Interventions classified by level of patient and family engagement.

Figure 2 provides a detailed overview of the level of patient engagement of the included interventions. Most remained at the Informing about engagement and prompting for engagement level (*n* = 13), a few were at the Empowerment level (*n* = 5), and one reached the Partnership level (Figure 2). There were proportionally more patient engagement interventions reaching levels 2 or 3 among study protocols and registrations (3/7, 43%) than among completed RCTs (3/12, 25%).

At the Informing about engagement and prompting level, four interventions consisted of informing patients or families about medication safety-related issues through oral, written, or multimedia documentation aimed at prompting discussions with care providers,^44^,^48^,^52^,^53^ two consisted of training care providers to engage in shared decision-making with patients,^56^,^61^ whereas the remaining seven provided opportunities for discussion about safety between patients/families and care providers.^46^,^47^,^49^–^51^,^58^,^60^

At the Empowerment level, interventions included communication tools to facilitate contact between patients and care providers^43^,^57^ and/or patient therapy or education and activation about safety-related issues,^45^,^55^,^57^,^59^ with one study including both.^57^

The Partnership level was reached by a single study protocol describing the use of patient-reported experience in patient safety planning at the care centre’s level^54^

All three interventions including family members were at level 1 engagement, and consisted of providing medication safety information to patients and families^48^,^53^ or organising multiple discussions with patients, care providers, and family and non-family carers about medication changes.^58^

Only one out of nine interventions targeting older adults reached level 2 engagement;^55^ all others remained at level 1. All interventions at level 2 engagement were from the US (*n* = 5), whereas the intervention protocol at level 3 engagement was from Spain.^54^ Four out of six interventions at engagement levels 2 or 3 (67%) were published before 2020.

### Results of individual studies

Whereas four trial registrations and study protocols with no corresponding record of a completed RCT were published since 2020,^46^,^52^,^54^,^61^ three were published between 2015 and 2019.^43^,^59^,^60^

Of the 12 completed RCTs, improvements were reported in three out of five focusing on adverse drug events,^44^,^45^,^47^ with only one of them being at level 2 engagement.^45^ Among the seven RCTs testing medication appropriateness, none found significant improvements, with only one reaching level 2 engagement.^57^ A single RCT, at level 2 engagement, investigated medication discrepancy and reported no improvement following the intervention.^59^ Of note, however, intervention and outcome in this study were not fully aligned, as the study defined patient and family education about the value of deprescribing as intervention, and correction of medication discrepancies as outcome.

Overall, only three interventions out of the 12 completed RCTs reported significant improvements in patient safety, of which two were targeting adults^44^,^45^ and one was targeting older adults.^47^ Although all three measured adverse drug events, one focused on antihypertensive drugs^44^ whereas the two others targeted a broader range of medications prone to causing falls or other adverse events.^45^,^47^

Eight RCTs demonstrated low risk of bias (Supplementary Figure S1), three raised some concern,^47^,^53^,^55^ whereas one RCT^45^ showed high overall risk of bias.

### Impact on specific outcomes

**Figure 3:**
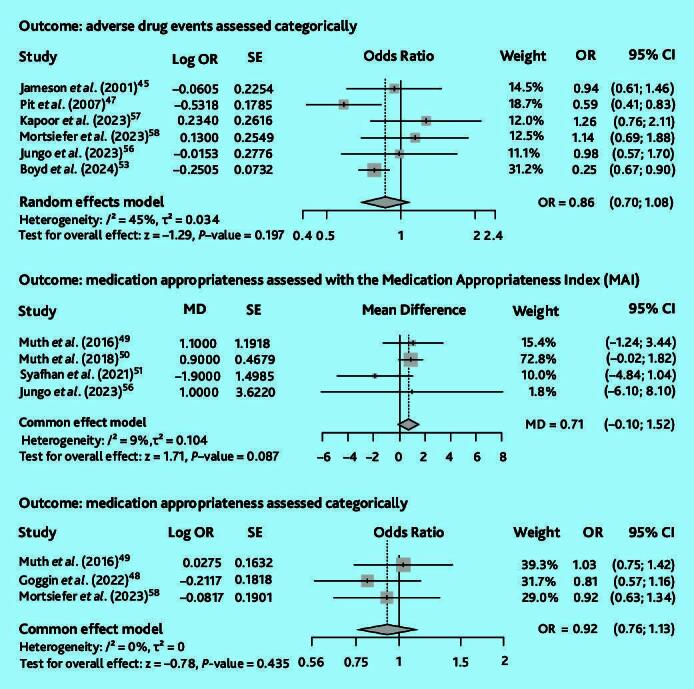
Meta-analyses of RCTs and cluster RCTs included in the review. CI = confidence interval. MD = mean difference. OR = odds ratio. RCT = randomised controlled trial. SE = standard error. Dotted vertical line and grey diamond = overall measure of effect.

Evidence from 10 out of 12 completed RCTs could be combined into three meta-analyses (Figure 3) to assess the outcomes related to adverse drug events (one meta-analysis of six studies) and medication appropriateness (two meta-analyses combining, respectively, four studies with continuous outcomes and three studies with categorical outcomes), with three studies included in two analyses each.^49^,^56^,^58^

The meta-analysis on adverse drug events (Figure 3, upper panel) excluded an RCT on the topic of promoting self-reporting of adverse drug events (with more reported adverse drug events considered more favourable),^43^ as the other RCTs assessed adverse drug events with more events considered less favourable.^45^,^47^,^53^,^56^–^58^ The combined analysis showed moderate heterogeneity (*I*^2^ = 45%) and no significant effect (OR 0.86, 95% confidence interval [CI] = 0.70 to 1.08, *P* = 0.197). Evidence certainty was rated as very low for this meta-analysis (Supplementary Table S7).

The meta-analysis of the four studies using the Medication Appropriateness Index scores to assess medication appropriateness continuously^49^–^51^,^56^ (Figure 3, middle panel) had low heterogeneity (*I*^2^ = 9%) and found no significant combined effect (mean difference 0.71, 95% CI = −0.10 to 1.52, *P* = 0.087). Evidence certainty was rated as moderate (Supplementary Table S7).

The meta-analysis of categorically measured medication appropriateness (counts of inappropriate prescriptions; Figure 3, lower panel) including three studies^48^,^49^,^58^ had low heterogeneity (*I*^2^ = 0%) and was also non-significant (OR 0.92, 95% CI = 0.76 to 1.13, *P* = 0.435), with moderate evidence certainty (Supplementary Table S7).

Ultimately, none of the combined effect of interventions yielded significant results, although trends suggested beneficial effects for all outcomes considered.

## Discussion

### Summary

The present review identified 19 interventions aimed at promoting patient and family engagement in the context of patient safety in primary care. All but one record focused on medication safety as an outcome. Meta-analyses did not yield significant combined effects, and only three out of 12 completed RCTs reported modest-to-moderate positive effects of patient and family engagement interventions individually, none of which included families.

### Strengths and limitations

The current review is the first, to the authors’ knowledge, to provide a comprehensive overview of randomised controlled interventions targeting patient and family engagement in primary care patient safety. It benefits from a comprehensive approach, including trial registrations and protocols, and a rigorous methodology adhering to the Cochrane Collaboration guidelines.

Despite the search strategy being based on existing systematic reviews on patient safety and refined with a university librarian’s guidance, it may have omitted keywords relevant to specific patient safety outcomes given the varying definitions of adverse events in primary care.^63^ The lack of a universally recognised definition of patient engagement might also have led to exclusions because of terminological variations. Additionally, the study’s focus on English-language, peer-reviewed RCTs may have omitted relevant records in other languages or less resource-expensive designs. Since the meta-analysis includes only completed RCTs further insights may arise from ongoing RCTs once completed. Finally, the study’s exclusion of interventions conducted in specialised care have precluded us from identifying interventions relevant to primary care but tested in other ambulatory care settings such as specialised care.

### Comparison with existing literature

The lack of patient engagement at the global care level aligns with a general scarcity of interventions fully integrating patients as team members in healthcare settings.^29^ Except for medication reconciliation, the evidence-based strategies of patient and family engagement recommended by the Agency for Healthcare Research and Quality^64^ were not investigated. Such strategies could have included being prepared to be engaged (patients and families encouraged to prepare for their appointments), teach-back (asking the patient/family to explain the instructions in their own words), and warm handoff (in-person handoff conducted in front of the patient).

Family involvement in the reported interventions remained limited to three studies, one of which occurred in a paediatric age group.^48^ Although the incorporation of family members introduces complexities in terms of study design, trials can be adapted to accommodate their needs, for example, by preparing separate study information materials and adapting surveys for family members. Meanwhile, family engagement remains a valuable resource in routine clinical practice with a demonstrated positive impact on patient–provider communication.^65^

Overall, the reported interventions had a considerable overlap with standard of care. Many of them offered one-time consultations or written information to help patients understand their health and enhance communication with the care team that, although potentially useful in identifying certain existing safety issues, may be insufficient or too short-term to provide lasting results, especially in the case of infrequent patient safety outcomes, such as falls. However, healthcare professionals can find the implementation of more complex interventions in the clinical workflow challenging, and/or could fail to consider the added value of higher levels of patient engagement for patient safety.^66^

This review underscores the dearth of research into safety outcomes in primary care beyond the scope of medication safety. In particular, errors linked to other aspects of primary care delivery, such as communication errors or errors associated with care management, may be important to target.^11^ Such errors occur at a high frequency,^67^ being estimated at four out of every 1000 primary care encounters.^68^ In qualitative studies, they appear in the patient safety conceptualisations of patients,^69^,^70^ but less clearly in those of care providers, who tend to primarily consider medication safety issues and professional or organisational shortcomings under patient safety.^71^,^72^ Mending this conceptual gap about patient safety may be key to devising patient safety interventions with higher levels of patient engagement. It may need to start with global efforts to modernise patient safety culture and promote patient–provider partnership among primary care providers.

### Implications for research and practice

Despite the potential of patient and family engagement in enhancing patient safety in primary care, the available evidence falls short of demonstrating unequivocal effectiveness. This is useful and actionable information for busy clinicians as knowing what does not work helps avoiding investing in efforts that will not provide any tangible benefits to the patient or the population served by the practice. This information will not only support busy clinicians in making necessary choices, but it should also prompt researchers to evaluate why these carefully assessed interventions have not worked in order to consider more promising approaches.

Future research and practice may need to think outside of the box of common patient safety outcomes such as medication safety and traditional patient involvement such as patient information to attain patient and family engagement in overall primary care patient safety. Patient and public involvement may be crucial in this respect, as patients have insightful and often different perspectives about safety determinants, the stages of patient safety they could be involved in at both individual and care centre levels, and about potential barriers to their involvement, such as missing knowledge, skills or tools, that an intervention could help them acquire.^73^

## Supplementary Information



## Data Availability

All relevant data from this study are available from the corresponding author.
