# Supplementary material for: Patient and family engagement interventions in primary care patient safety: systematic review and meta-analysis of randomised controlled trials
Source: Br J Gen Pract. 2025 Feb 18;75(756):e491–9. doi: 10.3399/BJGP.2024.0369 (PMC12364509; doi:10.3399/BJGP.2024.0369)
Supplement: Supplementary file 1 [file BJGP-2024-0369_Leong_Supplement.pdf]

SUPPLEMENT FOR *Patient and family engagement interventions in primary care patient safety: a systematic review and meta-analysis*

Complete search strategy

Supplemental Table S1 - Ovid MEDLINE

concepts		search terms as run	hits	source of search terms	change from original	reason
concept 1 primary care	1	exp General Practice/ or exp Ambulatory Care/ or exp Primary Health Care/ or (((primary or community) adj2 (care? or health$ or service?)) or (ambulatory adj2 (care or service))).tw.	5E+05	Ricci-Cabello 2015 https://doi.org/10.1093/fampra/cmu052		
	2	exp general practitioners/ or exp physicians, family/ or exp physicians, primary care/ or GP?.tw. or ((general adj1 (practitioner? or physician? or doctor?)) or (family adj1 (practitioner? or physician? or doctor? or nurse?)) or (primary adj1 physician?) or (community adj1 (practitioner? or physician? or doctor? or nurse? or provider?)) or (ambulatory adj1 (practitioner? or physician? or doctor? or nurs$3 or provider?))).tw.	2E+05	Ricci-Cabello 2015		
	3	exp community health centers/ or ((general or family or primary or community or ambulatory) adj1 (center? or centre? or practice?)).tw.	73551	Ricci-Cabello 2015		
	4	(AMERICAN FAMILY PHYSICIAN or AM FAM PHYSICIAN or "Am. Fam. Physician" or ANNALS OF FAMILY MEDICINE or ANN FAM MED or "Ann. Fam. Med" or Atencion Primaria or ATEN PRIM or "Aten. Prim." or BMC Family Practice or "BMC Fam. Pract." or BRITISH JOURNAL OF GENERAL PRACTICE or "Br. J. Gen. Pract." or BRIT J GEN PRACT or CANADIAN FAMILY PHYSICIAN or "Can. Fam. Phys." or CAN FAM PHYSICIAN or FAMILY MEDICINE or "Fam. Med" or "FAM MED." or FAMILY PRACTICE or "Fam. Pr." or "FAM PRACT." or Journal of the American Board of Family Medicine or "J. Am. Board Fam. Med." or J AM BOARD FAM MED or JOURNAL OF FAMILY PRACTICE or "J. Fam. Pract." or "J FAM PRACTICE." or "PRIMARY CARE." or SCANDINAVIAN JOURNAL OF PRIMARY HEALTH CARE or "Scand. J. Prim. Health Care" or PRIMARY CARE or Australian Family Physician or "Aust. Fam. Physician" or AUST FAM PHYSICIAN or Australian Journal of Primary Health or "Aust. J. Prim. Health" or AUST J PRIM HEALTH or European Journal of General Practice or "Eur. J. Gen. Pract." or npj Primary Care Respiratory Medicine or "NPJ Prim. Care Respir. Med." or "PHYSICIAN AND SPORTSMEDICINE" or Phys Sportsmed or Primary Care Diabetes or Prim Care Diabetes or Atencion Primaria or "Aten. Primaria." or "Primary Health Care Research and Development" or Prim Health Care Res Dev).jn.	82523	Ricci-Cabello 2015 & our own additions		
	5	1 or 2 or 3 or 4	7E+05	Ricci-Cabello 2015		
concept 2 patient safety	6	exp Safety Management/ or exp Patient Safety/ or exp Patient Harm/ or exp Medical Errors/ or patient safety.tw. or safety culture.tw. or (safe$2 adj2 (practice? or management?)).tw. or (st.fs. and (safe or safety).tw.)	2E+05	Ricci-Cabello 2015 additions based on the team's ongoing scoping review on patient and family engagement in patient safety		
	7	(((adverse? or preventable?) adj1 (event? or effect? or reaction? or outcome?)) or (safe$2 adj1 (event? or effect?))).tw.	5E+05	Ricci-Cabello 2015	original term: (((adverse? or preventable?) adj1 (event? or effect? or reaction? or outcome?)) or (safet$3 adj1 (event? or effect?))).tw. (456387 hits)	to include "safe", "safer", …; did not make sense to have three letters after "safet"; this change did not influence the number of hits
	8	harm$3.tw.	2E+05	Ricci-Cabello 2015	original term: harm$2.tw. (80249 hits)	to include "harmful"
	9	exp Iatrogenic Disease/ or iatrog$.tw.	1E+05	Ricci-Cabello 2015	changed iatrogenic to iatrog$	to include alternative endings of this word
	10	exp Medical Errors/ or ((medica$ or diagnos$ or therapeutic$ or administrat$ or dispensing? or prescri$ or clinical) adj1 (error? or mistake? or fault?)).tw.	1E+05	Ricci-Cabello 2015		
	11	exp Malpractice/ or malpractice?.tw. or negligen$.tw. or misconduct$.tw. or neglect$.tw.	1E+05	Ricci-Cabello 2015		
	12	("Quality & safety in health care" or Qual Saf Health Care or International Journal for Quality in Health Care or IJQHC).jn. and safe$2.mp.	1023	Ricci-Cabello 2015		
	13	6 or 7 or 8 or 9 or 10 or 11 or 12	1E+06	Ricci-Cabello 2015		
concept 3 patient & family engagement	14	exp Family/ or exp Patients/ or exp Interpersonal Relations/ or exp Health Education/ or exp Patient Acceptance of Health Care/ or exp Patient Care Team/	1E+06	Mackintosh et al. Interventions to increase patient and family involvement in escalation of care for acute life-threatening illness in community health and hospital settings. *Cochrane Database of Systematic Reviews 2020*, Issue 12. Art. No.: CD012829. https://doi.org/10.1002/14651858.CD012829.pub2		

	15	exp Community Participation/ or exp Patient Education as Topic/	1E+05	Hall J, Peat M, Birks Y, et al. Effectiveness of interventions designed to promote patient involvement to enhance safety: a systematic review Qual Saf Health Care (2010). https://doi.org/10.1136/qshc.2009.032748	1. We changed Family (this term only) and Patients (this term only) to Family (explode on all trees) and Patients (explode on all trees)<br>2. We did not include Decision Making (this term only); Decision Support Techniques (this term only); Health Facilities (explode on all trees); Critical Care (this term only); Critical Illness (this term only)	1. We wanted to include Mesh terms beneath Family (e.g., Family Support); we are only interested in Outpatients for this review<br>2. these terms were not relevant for our topic, which is different than the source paper's topic
	16	((patient$ or consumer$ or famil$ or relative$ or parent$ or child$ or women$ or carer$ or care?giver$ or care giver$ or advocate$ or public or lay or people or user$ or citizen$) adj5 (activat$ or involv$ or initiat$ or engag$ or participat$ or contribut$ or collaborat$ or role$ or cooperat$ or assist$ or champion$ or advoc$ or help-seek$ or view$ or attitude$ or role$ or contribut$ or engage$ or opinion$ or report$ or signal$ or consult$ or conducting or conducted)).tw.	2E+06	Mackintosh 2020<br>Hall 2010<br>our own additions<br>additions based on the team's ongoing scoping review on patient and family engagement in patient safety		
	17	partner$3.tw.	2E+05	Mackintosh 2020	We did not use the last part of this search term in the Mackintosh 2020 article search (in red): (((patient* or consumer* or family or families or relative* or parent* or child* or partner* or women* or carer* or caregiver* or advocate*) N5 (activat* or involv* or initiat* or engag* or participat* or contribut* or collaborat* or role or cooperat* or assist* or champion* or advoc* or help-seek*) N5 (deteriorat* or escalat* or "life threatening" or life-threatening or critical or emergenc* or complication* or "warning signs" or "danger signs" or adverse))):ti,ab,kw	irrelevant to our topic
	18	14 or 15 or 16 or 17	3E+06		separated this term from the previous entry	partner; partnering can be both a noun and a verb
concept 4 randomized - controlled trial	19	(controlled clinical trial or randomized controlled trial).pt. or (randomi?ed and controlled and trial).tw.	8E+05	Combines:<br>[1] filter with the highest f- score to detect randomized controlled trials according to this publication: https://www.ncbi.nlm.nih.gov/pmc/articles/PMC7524635/<br>- Nwosu et al. 1998 and<br>[2] PubMed filter "Narrow filter for randomized clinical trials" (https://pubmed.ncbi.nlm.nih.gov/help/#clinical-study-categories-bibliography)<br>extended to British spelling	randomized has been changed to randomi?ed	extend search term to British spelling
Concepts 1, 2, and 3 combined	20	5 and 13 and 18	13494			
**All concepts combined**	**21**	**20 and 19**	**1340**			

Supplemental Table S2 - Embase

concepts		search terms as run
concept 1 primary care	1	General Practice'/exp OR 'Ambulatory Care'/exp OR 'Primary Health Care'/exp OR (((primary OR community) NEAR/2 (care? OR health* OR service?)) OR (ambulatory NEAR/2 (care OR service))):ab,ti
	2	general practitioners'/exp OR 'physicians, family'/exp OR 'physicians, primary care'/exp OR GP?:ab,ti OR ((general NEAR/1 (practitioner? OR physician? OR doctor?)) OR (family NEAR/1 (practitioner? OR physician? OR doctor? OR nurse?)) OR (primary NEAR/1 physician?) OR (community NEAR/1 (practitioner? OR physician? OR doctor? OR nurse? OR provider?)) OR (ambulatory NEAR/1 (practitioner? OR physician? OR doctor? OR nurs* OR provider?))):ab,ti
	3	community health centers'/exp OR ((general OR family OR primary OR community OR ambulatory) NEAR/1 (center? OR centre? OR practice?)):ab,ti
	4	('AMERICAN FAMILY PHYSICIAN' OR 'AM FAM PHYSICIAN' OR 'Am. Fam. Physician' OR 'ANNALS OF FAMILY MEDICINE' OR 'ANN FAM MED' OR 'Ann. Fam. Med' OR 'Atencion Primaria' OR 'ATEN PRIM' OR 'Aten. Prim.' OR 'BMC Family Practice' OR 'BMC Fam. Pract.' OR 'BRITISH JOURNAL OF GENERAL PRACTICE' OR 'Br. J. Gen. Pract.' OR 'BRIT J GEN PRACT' OR 'CANADIAN FAMILY PHYSICIAN' OR 'Can. Fam. Phys.' OR 'CAN FAM PHYSICIAN' OR 'FAMILY MEDICINE' OR 'Fam. Med' OR 'FAM MED.' OR 'FAMILY PRACTICE' OR 'Fam. Pr.' OR 'FAM PRACT.' OR 'Journal of the American Board of Family Medicine' OR 'J. Am. Board Fam. Med.' OR 'J AM BOARD FAM MED' OR 'JOURNAL OF FAMILY PRACTICE' OR 'J. Fam. Pract.' OR 'J FAM PRACTICE.' OR 'PRIMARY CARE.' OR 'SCANDINAVIAN JOURNAL OF PRIMARY HEALTH CARE' OR 'Scand. J. Prim. Health Care' OR 'PRIMARY CARE' OR 'Australian Family Physician' OR 'Aust. Fam. Physician' OR 'AUST FAM PHYSICIAN' OR 'Australian Journal of Primary Health' OR 'Aust. J. Prim. Health' OR 'AUST J PRIM HEALTH' OR 'European Journal of General Practice' OR 'Eur. J. Gen. Pract.' OR 'npj Primary Care Respiratory Medicine' OR 'NPJ Prim. Care Respir. Med.' OR 'PHYSICIAN AND SPORTSMEDICINE' OR 'Phys Sportsmed' OR 'Primary Care Diabetes' OR 'Prim Care Diabetes' OR 'Aten. Primaria.' OR 'Primary Health Care Research AND Development' OR 'Prim Health Care Res Dev')/jt
	5	1 OR 2 OR 3 OR 4
concept 2 patient safety	6	Safety Management'/exp OR 'Patient Safety'/exp OR 'Patient Harm'/exp OR 'Medical Errors'/exp OR 'patient safety':ab,ti OR 'safety culture':ab,ti OR (safe* NEAR/2 (practice? OR management?)):ab,ti
	7	(((adverse? OR preventable?) NEAR/1 (event? OR effect? OR reaction? OR outcome?)) OR (safe* NEAR/1 (event? OR effect?))):ab,ti
	8	harm*:ab,ti
	9	Iatrogenic Disease'/exp OR iatrog*:ab,ti
	10	Medical Errors'/exp OR ((medica* OR diagnos* OR therapeutic* OR administrat* OR dispensing? OR prescri* OR clinical) NEAR/1 (error? OR mistake? OR fault?)):ab,ti
	11	Malpractice'/exp OR malpractice?:ab,ti OR negligen*:ab,ti OR misconduct*:ab,ti OR neglect*:ab,ti
	12	('Quality & safety in health care' OR 'Qual Saf Health Care' OR 'International Journal for Quality in Health Care' OR IJQHC)/jt AND safe*:ab,ti
	13	6 OR 7 OR 8 OR 9 OR 10 OR 11 OR 12
concept 3 patient & family engagement	14	family'/exp OR 'patients'/exp OR 'interpersonal relations'/exp OR 'health education'/exp OR 'patient acceptance of health care'/exp OR 'patient care team'/exp
	15	Community Participation'/exp OR 'Patient Education as Topic'/exp
	16	((patient* OR consumer* OR famil* OR relative* OR parent* OR child* OR women* OR carer* OR care?giver* OR 'care giver' OR 'care givers' OR advocate* OR public OR lay OR people OR user* OR citizen*) NEAR/5 (activat* OR involv* OR initiat* OR engag* OR participat* OR collaborat* OR cooperat* OR assist* OR champion* OR advoc* OR help?seek* OR view* OR attitude* OR role* OR contribut* OR engage* OR opinion* OR report* OR signal* OR consult* OR conducting OR conducted)):ab,ti
	17	partner*:ab,ti
	18	14 OR 15 OR 16 OR 17
concept 4 randomized-controlled trial	19	('controlled clinical trial' OR 'randomized controlled trial'):pt OR (randomi?ed AND controlled AND trial):ab,ti
Concepts 1, 2, and 3 combined	20	5 AND 13 AND 18
**All concepts combined**	**21**	**20 AND 19**

Supplemental Table S3 - Web of Science

concepts		search terms as run
concept 1<br>primary care	1	TS=(((primary OR community) NEAR/2 (care? OR health* OR service?)) OR (ambulatory NEAR/2 (care OR service)))
	2	TS=(GP OR GPs OR (general NEAR/1 (practitioner? OR physician? OR doctor?)) OR (family NEAR/1 (practitioner? OR physician? OR doctor? OR nurse?)) OR (primary NEAR/1 physician?) OR (community NEAR/1 (practitioner? OR physician? OR doctor? OR nurse? OR provider?)) OR (ambulatory NEAR/1 (practitioner? OR physician? OR doctor? OR nurs* OR provider?)))
	3	TS=((general OR family OR primary OR community OR ambulatory) NEAR/1 (center? OR centre? OR practice?))
	4	SO=(('AMERICAN FAMILY PHYSICIAN' OR 'AM FAM PHYSICIAN' OR 'Am. Fam. Physician' OR 'ANNALS OF FAMILY MEDICINE' OR 'ANN FAM MED' OR 'Ann. Fam. Med' OR 'Atencion Primaria' OR 'ATEN PRIM' OR 'Aten. Prim.' OR 'BMC Family Practice' OR 'BMC Fam. Pract.' OR 'BRITISH JOURNAL OF GENERAL PRACTICE' OR 'Br. J. Gen. Pract.' OR 'BRIT J GEN PRACT' OR 'CANADIAN FAMILY PHYSICIAN' OR 'Can. Fam. Phys.' OR 'CAN FAM PHYSICIAN' OR 'FAMILY MEDICINE' OR 'Fam. Med' OR 'FAM MED.' OR 'FAMILY PRACTICE' OR 'Fam. Pr.' OR 'FAM PRACT.' OR 'Journal of the American Board of Family Medicine' OR 'J. Am. Board Fam. Med.' OR 'J AM BOARD FAM MED' OR 'JOURNAL OF FAMILY PRACTICE' OR 'J. Fam. Pract.' OR 'J FAM PRACTICE.' OR 'PRIMARY CARE.' OR 'SCANDINAVIAN JOURNAL OF PRIMARY HEALTH CARE' OR 'Scand. J. Prim. Health Care' OR 'PRIMARY CARE' OR 'Australian Family Physician' OR 'Aust. Fam. Physician' OR 'AUST FAM PHYSICIAN' OR 'Australian Journal of Primary Health' OR 'Aust. J. Prim. Health' OR 'AUST J PRIM HEALTH' OR 'European Journal of General Practice' OR 'Eur. J. Gen. Pract.' OR 'npj Primary Care Respiratory Medicine' OR 'NPJ Prim. Care Respir. Med.' OR 'PHYSICIAN AND SPORTSMEDICINE' OR 'Phys Sportsmed' OR 'Primary Care Diabetes' OR 'Prim Care Diabetes' OR 'Aten. Primaria.' OR 'Primary Health Care Research AND Development' OR 'Prim Health Care Res Dev'))
	5	1 OR 2 OR 3 OR 4
concept 2<br>patient safety	6	TS=(patient safety OR safety culture OR (safe* NEAR/2 (practice? OR management?)))
	7	TS=(((adverse? OR preventable?) NEAR/1 (event? OR effect? OR reaction? OR outcome?)) OR (safe* NEAR/1
	8	TS=harm*
	9	TS= iatrog*
	10	TS=(((medica* OR diagnos* OR therapeutic* OR administrat* OR dispensing? OR prescri* OR clinical) NEAR/1 (error? OR mistake? OR fault?)))
	11	TS=(malpractice?:ab,ti OR negligen$:ab,ti OR misconduct$:ab,ti OR neglect$)
	12	SO=('Quality & safety in health care' OR Qual Saf Health Care OR International Journal for Quality in Health Care OR IJQHC) AND TS=safe*
	13	6 OR 7 OR 8 OR 9 OR 10 OR 11 OR 12
concept 3<br>patient & family engagement	14	*Mesh terms - not applicable*
	15	*Mesh terms - not applicable*
	16	TS=((patient* OR consumer* OR famil* OR relative* OR parent* OR child* OR women* OR carer* OR care?giver* OR advocate* OR public OR lay OR people OR user* OR citizen*) NEAR/5 (activat* OR involv* OR initiat* OR engag* OR participat* OR collaborat* OR cooperat* OR assist* OR champion* OR advoc* OR help?seek* OR view* OR attitude* OR role* OR contribut* OR engage* OR opinion* OR report* OR signal* OR consult* OR conducting OR conducted))
	17	TS=partner*
	18	14 OR 15 OR 16 OR 17
concept 4<br>randomized-controlled trial	19	TS=(('controlled clinical trial' OR 'randomized controlled trial') OR (randomi?ed AND controlled AND trial))
Concepts 1, 2, and 3 combined	20	5 AND 13 AND 18
**All concepts combined**	**21**	**20 AND 19**

Supplemental Table S4 - Cochrane

concepts		search terms as run
concept 1 primary care	1	[mh "General Practice"] OR [mh "Ambulatory Care"] OR [mh "Primary Health Care"] OR (((primary OR community) NEAR/2 (care? OR health* OR service?)) OR (ambulatory NEAR/2 (care OR service))):ab,ti
	2	[mh "general practitioners"] OR [mh "physicians, family"] OR [mh "physicians, primary care"] OR GP?:ab,ti OR ((general NEAR/1 (practitioner? OR physician? OR doctor?)) OR (family NEAR/1 (practitioner? OR physician? OR doctor? OR nurse?)) OR (primary NEAR/1 physician?) OR (community NEAR/1 (practitioner? OR physician? OR doctor? OR nurse? OR provider?)) OR (ambulatory NEAR/1 (practitioner? OR physician? OR doctor? OR nurs* OR provider?))):ab,ti
	3	[mh "community health centers"] OR ((general OR family OR primary OR community OR ambulatory) NEAR/1 (center? OR centre? OR practice?)):ab,ti
	4	*not applicable*
	5	1 OR 2 OR 3
concept 2 patient safety	6	[mh "Safety Management"] OR [mh "Patient Safety"] OR [mh "Patient Harm"] OR [mh "Medical Errors"] OR "patient safety":ab,ti OR "safety culture":ab,ti OR (safe* NEAR/2 (practice? OR management?)):ab,ti
	7	(((adverse? OR preventable?) NEAR/1 (event? OR effect? OR reaction? OR outcome?)) OR (safe* NEAR/1 (event? OR effect?))):ab,ti
	8	harm*:ab,ti
	9	[mh "Iatrogenic Disease"] OR iatrog*:ab,ti
	10	[mh "Medical Errors"] OR ((medica* OR diagnos* OR therapeutic* OR administrat* OR dispensing? OR prescri* OR clinical) NEAR/1 (error? OR mistake? OR fault?)):ab,ti
	11	[mh "Malpractice"] OR malpractice?:ab,ti OR negligen*:ab,ti OR misconduct*:ab,ti OR neglect*:ab,ti
	12	*not applicable*
	13	6 OR 7 OR 8 OR 9 OR 10 OR 11 OR 12
concept 3 patient & family engagement	14	[mh "family"] OR [mh "patients"] OR [mh "interpersonal relations"] OR [mh "health education"] OR [mh "patient acceptance of health care"] OR [mh "patient care team"]
	15	[mh "Community Participation"] OR [mh "Patient Education as Topic"]
	16	((patient* OR consumer* OR famil* OR relative* OR parent* OR child* OR women* OR carer* OR care?giver* OR "care giver" OR "care givers" OR advocate* OR public OR lay OR people OR user* OR citizen*) NEAR/5 (activat* OR involv* OR initiat* OR engag* OR participat* OR collaborat* OR cooperat* OR assist* OR champion* OR advoc* OR help?seek* OR view* OR attitude* OR role* OR contribut* OR engage* OR opinion* OR report* OR signal* OR consult* OR conducting OR conducted)):ab,ti
	17	partner*:ab,ti
	18	14 OR 15 OR 16 OR 17
concept 4 randomized-controlled trial	19	("controlled clinical trial" OR "randomized controlled trial").pt. OR (randomi?ed AND controlled AND trial):ab,ti
Concepts 1, 2, and 3 combined	20	5 AND 13 AND 18
**All concepts combined**	**21**	**20 AND 19**

Supplemental Table S5 - CINHAL

concepts		search terms as run
concept 1 primary care	1	MH "General Practice+" OR MH "Ambulatory Care+" OR MH "Primary Health Care+" OR (((primary OR community) N2 (care# OR health* OR service#)) OR (ambulatory N2 (care OR service))):ab,ti
	2	MH "general practitioners+" OR MH "physicians, family+" OR MH "physicians, primary care+" OR GP#:ab,ti OR ((general N1 (practitioner# OR physician# OR doctor#)) OR (family N1 (practitioner# OR physician# OR doctor# OR nurse#)) OR (primary N1 physician#) OR (community N1 (practitioner# OR physician# OR doctor# OR nurse# OR provider#)) OR (ambulatory N1 (practitioner# OR physician# OR doctor# OR nurs* OR provider#))):ab,ti
	3	MH "community health centers+" OR ((general OR family OR primary OR community OR ambulatory) N1 (center# OR centre# OR practice#)):ab,ti
	4	*not applicable*
	5	1 OR 2 OR 3
concept 2 patient safety	6	MH "Safety Management+" OR MH "Patient Safety+" OR MH "Patient Harm+" OR MH "Medical Errors+" OR "patient safety":ab,ti OR "safety culture":ab,ti OR (safe* N2 (practice? OR management#)):ab,ti
	7	(((adverse# OR preventable#) N1 (event# OR effect# OR reaction# OR outcome#)) OR (safe* N1 (event# OR effect#))):ab,ti
	8	harm*:ab,ti
	9	MH "Iatrogenic Disease+" OR iatrog*:ab,ti
	10	MH "Medical Errors+" OR ((medica* OR diagnos* OR therapeutic* OR administrat* OR dispensing# OR prescri* OR clinical) N1 (error# OR mistake# OR fault#)):ab,ti
	11	MH "Malpractice+" OR malpractice#:ab,ti OR negligen*:ab,ti OR misconduct*:ab,ti OR neglect*:ab,ti
	12	*not applicable*
	13	6 OR 7 OR 8 OR 9 OR 10 OR 11 OR 12
concept 3 patient & family engagement	14	MH "family+" OR MH "patients+" OR MH "interpersonal relations+" OR MH "health education+" OR MH "patient acceptance of health care+" OR MH "patient care team+"
	15	MH "Community Participation+" OR MH "Patient Education as Topic+"
	16	((patient* OR consumer* OR famil* OR relative* OR parent* OR child* OR women* OR carer* OR care#giver* OR "care giver" OR "care givers" OR advocate* OR public OR lay OR people OR user* OR citizen*) N5 (activat* OR involv* OR initiat* OR engag* OR participat* OR collaborat* OR cooperat* OR assist* OR champion* OR advoc* OR help#seek* OR view* OR attitude* OR role* OR contribut* OR engage* OR opinion* OR report* OR signal* OR consult* OR conducting OR conducted)):ab,ti
	17	partner*:ab,ti
	18	14 OR 15 OR 16 OR 17
concept 4 randomized-controlled trial	19	("controlled clinical trial" OR "randomized controlled trial").pt. OR (randomi?ed AND controlled AND trial):ab,ti
Concepts 1, 2, and 3 combined	20	5 AND 13 AND 18
**All concepts combined**	**21**	**20 AND 19**

Supplemental Figure S1: Risk of bias assessment of included cluster RCTs and RCTs

Intention-to-treat

Study ID	D1a<br>Randomisation process	D1b<br>Timing of identification or recruitment of participants	D2<br>Deviations from the intended interventions	D3<br>Missing outcome data	D4<br>Measurement of the outcome	D5<br>Measurement of the outcome	Overall
Buchet-Poyau 2021	+	+	+	+	+	+	+
Boyd 2024	+	!	+	+	+	+	!
Goggin 2022	+	+	+	+	+	+	+
Jungo 2023	+	+	+	+	+	+	+
Mortsiefer 2023	+	+	+	+	+	+	+
Muth 2016	+	+	+	+	+	+	+
Muth 2018	+	+	+	+	+	+	+
Pit 2007	+	+	!	!	+	+	!
Fried 2017	+	NA	!	+	+	+	!
Jameson 2001	+	NA	-	+	+	+	-
Kapoor 2023	+	NA	+	+	+	+	+
Syafhan 2021	+	NA	+	+	+	+	+

+ Low risk
! Some concerns
- High risk

Note. Concerns regarding risk of bias primarily arose due to either significant deviations from the intended interventions or the presence of missing outcome data.

Supplemental Table S7 Patient and family engagement compared to standard of care to improve patient safety

Certainty assessment							№ of patients		Effect		Certainty
№ of studies	Study design	Risk of bias	Inconsistency	Indirectness	Imprecision	Other considerations	patient involvement	usual care	Relative (95% CI)	Absolute (95% CI)	
**Adverse drug events (follow-up: median 6 months)**											
6[1,2,3,4,5,6]	randomised trials	serious[a]	not serious	serious[b]	serious[c]	none	1125/2650 (42.5%)	1289/2720 (47.4%)	**OR 0.86** (0.70 to 1.08)	**37 fewer per 1000** (from 87 fewer to 19 more)	⊕◯◯◯ Very low[a,b,c]
**Inappropriate prescription (number of patients receiving an inappropriate prescription) (follow-up: mean 8 weeks)**											
3[3,7,8]	randomised trials	not serious	not serious	not serious	serious[c]	none	337/1352 (24.9%)	355/1523 (23.3%)	**OR 0.92** (0.76 to 1.13)	**15 fewer per 1000** (from 45 fewer to 23 more)	⊕⊕⊕◯ Moderate[c]
**Medication appropriateness index (MAI score) (follow-up: median 6 months)**											
4[4,7,9,10]	randomised trials	not serious	not serious	not serious	serious[c]	none	347	335	-	MD **0 0.71** (0.1 lower to 1.52 higher)	⊕⊕⊕◯ Moderate[c]
**Medication disprepency correction (follow-up: mean 3 months)**											
1[11]	randomised trials	serious[d]	not serious	not serious	not serious	none	No differences between intervention and control patients in the number of medications prescribed at ninety days or in the number of TRIM-related recommendations implemented<br>However, over three times as many patients who received the intervention had correction of medication reconciliation errors as those who did not (48.4% versus 14.3%, p < .001)				⊕⊕⊕◯ Moderate[d]

CI: confidence interval; **MD:** mean difference; **OR:** odds ratio

Explanations

a. Pit 2007 presents some concerns due to deviations from the intended interventions and missing outcome data. Jameson 2001 presents a high risk of bias due to deviations from the intended intervention. The remaining three trials presented low risk of bias. Boyd 2024 presented issues related to timing of identification/recruitment of participants
b. Falls reported as a proxy of adverse drug events in Pit 2007 and Jungo 2023.
c. Wide confidence intervals
d. Some concerns due to deviations from the intended intervention

References

1.Pit, S. W., Byles, J. E., Henry, D. A., Holt, L., Hansen, V., Bowman, D. A.. A Quality Use of Medicines program for general practitioners and older people: a cluster randomised controlled trial.Med J Aust; Jul 2 2007.
2.Jameson, J. P., VanNoord, G. R.. Pharmacotherapy consultation on polypharmacy patients in ambulatory care.Ann Pharmacother; Jul-Aug 2001.
3.Mortsiefer, A., Löscher, S., Pashutina, Y., Santos, S., Altiner, A., Drewelow, E., Ritzke, M., Wollny, A., Thürmann, P., Bencheva, V., Gogolin, M., Meyer, G., Abraham, J., Fleischer, S., Icks, A., Montalbo, J., Wiese, B., Wilm, S., Feldmeier, G.. Family Conferences to Facilitate Deprescribing in Older Outpatients With Frailty and With Polypharmacy: The COFRAIL Cluster Randomized Trial.JAMA Netw Open; Mar 1 2023.
4.Jungo, K. T., Ansorg, A. K., Floriani, C., Rozsnyai, Z., Schwab, N., Meier, R., Valeri, F., Stalder, O., Limacher, A., Schneider, C., Bagattini, M., Trelle, S., Spruit, M., Schwenkglenks, M., Rodondi, N., Streit, S.. Optimising prescribing in older adults with multimorbidity and polypharmacy in primary care (OPTICA): cluster randomised clinical trial.Bmj; May 24 2023.
5.Kapoor, A., Patel, P., Mbusa, D., Pham, T., Cicirale, C., Tran, W., Beavers, C., Javed, S., Wagner, J., Swain, D., Crawford, S., Darling, C., ItoFuKunaga, M., McManus, D., Mazor, K., Gurwitz, J.. Multicomponent Pharmacist Intervention Did Not Reduce Clinically Important Medication Errors for Ambulatory Patients Initiating Direct Oral Anticoagulants.J Gen Intern Med; Dec 2023.
6.Boyd, C. M., Shetterly, S. M., Powers, J. D., Weffald, L. A., Green, A. R., Sheehan, O. C., Reeve, E., Drace, M. L., Norton, J. D., Maiyani, M., Gleason, K. S., Sawyer, J. K., Maciejewski, M. L., Wolff, J. L., Kraus, C., Bayliss, E. A.. Evaluating the Safety of an Educational Deprescribing Intervention: Lessons from the Optimize Trial.Drugs Aging; Jan 2024.
7.Muth, C., Harder, S., Uhlmann, L., Rochon, J., Fullerton, B., Güthlin, C., Erler, A., Beyer, M., van den Akker, M., Perera, R., Knottnerus, A., Valderas, J. M., Gerlach, F. M., Haefeli, W. E.. Pilot study to test the feasibility of a trial design and complex intervention on PRIoritising MUltimedication in Multimorbidity in general practices (PRIMUMpilot).BMJ Open; Jul 25 2016.
8.Goggin, K., Hurley, E. A., Lee, B. R., Bradley-Ewing, A., Bickford, C., Pina, K., Donis de Miranda, E., Yu, D., Weltmer, K., Linnemayr, S., Butler, C. C., Newland, J. G., Myers, A. L.. Let's Talk About Antibiotics: a randomised trial of two interventions to reduce antibiotic misuse.BMJ Open; Nov 21 2022.
9.Syafhan, N. F., Al Azzam, S., Williams, S. D., Wilson, W., Brady, J., Lawrence, P., McCrudden, M., Ahmed, M., Scott, M. G., Fleming, G., Hogg, A., Scullin, C., Horne, R., Ahir, H., McElnay, J. C.. General practitioner practice-based pharmacist input to medicines optimisation in the UK: pragmatic, multicenter, randomised, controlled trial.J Pharm Policy Pract; Jan 4 2021.
10.Muth, C., Uhlmann, L., Haefeli, W. E., Rochon, J., van den Akker, M., Perera, R., Güthlin, C., Beyer, M., Oswald, F., Valderas, J. M., Knottnerus, J. A., Gerlach, F. M., Harder, S.. Effectiveness of a complex intervention on Prioritising Multimedication in Multimorbidity (PRIMUM) in primary care: results of a pragmatic cluster randomised controlled trial.BMJ Open; Feb 24 2018.
11.Fried, T. R., Niehoff, K. M., Street, R. L., Charpentier, P. A., Rajeevan, N., Miller, P. L., Goldstein, M. K., O'Leary, J. R., Fenton, B. T.. Effect of the Tool to Reduce Inappropriate Medications on Medication Communication and Deprescribing.J Am Geriatr Soc; Oct 2017.

Supplemental Table S6: Study characteristics

Author, year	Type of record	Country/setting	Interventions[a] and their level of patient/family engagement	Study groups (N per group; control intervention)	Patient safety outcome[b]	Longest follow-up duration	Results pertaining to patient/family engagement in patient safety	Related publication (protocol, trial registration)
Outcome: adverse drug events (ADE)								
Abdel Shaheed, C., et al.(2023)	Cluster RCT Protocol	Australia/general practice	Interactive pamphlet as support for patients to ask GP about medications and pain management options<br><br>*Level 1: Informing about engagement and prompting for engagement*	I: 305<br>C:305<br>Standard care	Hospitalisations; Opioid-related poisonings; death<br><br>Medications: Opioids	52 weeks	NA	Trial registration: https://anzctr.org.au/Trial/Registration/TrialReview.aspx?ACTRN=12622001505796
Bohnert (2015)	RCT trial registration	USA/primary care clinic	Motivational & cognitive behavioural therapy on reducing reliance on opioids<br><br>*Level 2: Empowerment*	I: 225<br>C: 225<br>Enhanced usual care (educational content on the biology of pain & overview of pain conditions)	Patients with non-fatal overdose experiences or level of oversedation as an opioid side effect<br><br>Medications: Opioid/ Morphine	12 months	NA	Trial registration: https://clinicaltrials.gov/study/NCT02464410
Boyd,C.M., et al.(2024)[d]	Cluster RCT	USA/primary care clinics	Mailed information to patients/families regarding medication safety-related issues to prompt discussion with GP<br><br>*Level 1: Informing about engagement and prompting for engagement*	I: 1433; C: 1579 Standard care	Mortality; Hospitalization; Emergency Department visits<br><br>Medications: chronic medicine	8 months	No effect on hospitalization, mortality, or ED visits (*p*-values not reported)<br><br>Mortality:<br>Number of deaths:<br>I: 62/1433; C 59/1579<br><br>Number of hospitalisations:<br>I: 420/1433; C:517/1579<br><br>Number of Emergency Department visits:<br>I: 230/1433; C:307/1579	Trial registration: https://clinicaltrials.gov/study/NCT03984396<br><br>Published protocol: Bayliss et al. 2020 (https://doi.org/10.1186/s13063-020-04482-0)

Author, year	Type of record	Country/setting	Interventions[a] and their level of patient/family engagement	Study groups (N per group; control intervention)	Patient safety outcome[b]	Longest follow-up duration	Results pertaining to patient/family engagement in patient safety	Related publication (protocol, trial registration)
Buchet-Poyau, K., et al. (2021)	Cluster RCT	France/general practitioner clinics	Interactive patient booklet as support for discussion with GP<br><br>*Level 1: Informing about engagement and prompting for engagement*	I: 546<br>C: 549<br>Standard care	Number of patient reporting on ADE<br><br>Medications: Antihypertensive drugs (drug names were not provided)	3 months	Increase in reporting of adverse drug events<br><br>No. of patients reporting ADEs: I:57/546; C:34/549.<br><br>OR=3.9, 95% CI [1.4-11.2], *p*=0.01<br><br>aOR=3.5, 95%CI [1.2-10.1], *p*=0.02 (adjusted for age, sex, education, morbidity, ADE risk presence, antihypertensive prescription)	Trial registration: https://clinicaltrials.gov/study/NCT01610817<br><br>Published protocol: Keriel-Gascou et al. 2013 https://doi.org/10.1186/1748-5908-8-69
Cooper Bailey, S., et al. (2016)	Cluster RCT protocol	USA/ambulatory care	Electronic medication communication tool to facilitate contact between patients and physicians about drug safety<br><br>*Level 2: Empowerment*	I: 480<br>C: 480<br>Standard care	ADE<br><br>Medications: 69 higher-risk medications selected (not listed specifically)	3 months	NA	Trial registration: https://clinicaltrials.gov/study/NCT02785458
Jameson, J. P. and G. R. VanNoord (2001)	RCT	USA/physician practice clinics	Assessment of polypharmacy patients' understanding of medications & psychoeducation<br><br>*Level 2: Empowerment*	I: 179<br>C:161<br>Standard care	ADE: combination of 18 symptoms commonly attributed to ADEs<br><br>Medications: β-blocker, Warfarin, Steroid inhaler, Levothyroxine, Lithium, Allopurinol, Flurazepam, Glipizide	6 months	Improvement of symptoms attributed to ADEs<br><br>Participants reporting symptom improvement (only per protocol statistics reported): I:67/121, C:58/144, *p*=0.024 (chi-square statistics not reported)	NA

Author, year	Type of record	Country/setting	Interventions[a] and their level of patient/family engagement	Study groups (N per group; control intervention)	Patient safety outcome[b]	Longest follow-up duration	Results pertaining to patient/family engagement in patient safety	Related publication (protocol, trial registration)
Jungo, K.T., et al.(2023)	Cluster RCT	Switzerland/ general practice	Training course for primary care providers on how to employ shared decision-making strategies to promote patient engagement<br><br>*Level 1: Informing about engagement and prompting for engagement*	I: 160<br>C:163<br>Standard care	Medication Appropriateness; falls, fractures[c]<br><br>Medications:<br>Not specified	12 months	No effect on medication appropriateness, falls and fractures at 12 months<br><br>Participants with improved MAI:<br>I:68/160; C:67/163<br>OR = 1.05; 95%CI [0.59; 1.87] p=0.87<br><br>Mean no. of falls:<br>I: 0.2; 95%CI [0.1;0.3]<br>C: 0.2; 95%CI [0.1;0.3];<br>Incidence Rate Ratio (IRR) = 0.90; 95%CI [0.50 ; 1.64] p=0.74<br><br>Participants with any fractures:<br>I:3/160; C:2/163<br>OR = 1.51; 95%CI [0.27; 8.50] p=0.64	Trial registration: https://clinicaltrials.gov/study/NCT03724539<br><br>Published protocol:<br>Jungo et al. 2019 (http://dx.doi.org/10.1136/bmjopen-2019-031080)
Mangin, D., et al. (2021)	RCT protocol	Canada/primary care clinics	Patients asked to give feedback about medication intake and related problems<br><br>*Level 1: Informing about engagement and prompting for engagement*	I: 180<br>C:180<br>Waitlist control	Fall and other serious adverse events<br><br>Medications:<br>not specified	6 months	NA	Trial registration: https://clinicaltrials.gov/study/NCT02942927

Author, year	Type of record	Country/setting	Interventions[a] and their level of patient/family engagement	Study groups (N per group; control intervention)	Patient safety outcome[b]	Longest follow-up duration	Results pertaining to patient/family engagement in patient safety	Related publication (protocol, trial registration)
Pit, S. W., et al. (2007)	Cluster RCT	Australia/general practices	Patients completing medication risk assessment with their doctor<br><br>*Level 1: Informing about engagement and prompting for engagement*	I: 452<br>C: 397<br>Completed a Medication Risk Assessment forms but did not pass to GPs	Specific ADEs: slipped, tripped or stumbled OR falls OR fainted, felt weak or dizzy[c]<br><br>Medications:<br>Benzodiazepines, NSAIDs (including COX-2 inhibitors), Thiazide diuretics	12 months	Decrease in reported fall events in the intervention group.<br><br>No. of patients reporting fall events at 12 months (only per protocol statistics reported):<br>I:70/350, C:94/309.<br><br>OR (95%CI): 0.57 (0.40–0.81)<br>p=0.004;<br><br>aOR (95% CI): 0.61 (0.41–0.91)<br>p=0.02<br>(adjusted for age, sex, general practice, baseline event score, quality of life as per SF-12 score)	Trial registration: https://anzctr.org.au/Trial/Registration/TrialReview.aspx?ACTRN=012605000264684
Weiss,K., et al.(2023)	Cluster RCT protocol	Switzerland/ ambulatory primary care	Training course for primary care providers on how to employ shared decision-making strategies to promote patient engagement in medication safety<br><br>*Level 1: Informing about engagement and prompting for engagement*	I:184<br>C:184<br>Control group GP to get online training including advice on discussing general sleep hygiene	Falls<br><br>Medications:<br>Benzodiazepines and other sedative hypnotics	14 months	NA	Trial registration: https://www.isrctn.com/ISRCTN34363838
Outcome: medication appropriateness								
Goggin, K., et al. (2022)[d]	Cluster RCT	USA/private practice outpatient clinics	Video & brochure for parents on antibiotic misuse in children & encouraged to discuss issues with GP<br><br>*Level 1: Informing about*	I: 697<br>C: 904<br>Lower intensity intervention group and usual care	Patients receiving an inappropriate antibiotic prescription<br><br>Medications:<br>Amoxicillin Cefdinir, cefpodoxime,	2 weeks	No impact on inappropriate antibiotic prescription<br><br>Participants with inappropriate antibiotic prescription (only per protocol statistics reported):<br>I: 54/696, C: 85/903,	Trial registration: https://clinicaltrials.gov/study/NCT03037112<br><br>Published protocol:

Author, year	Type of record	Country/setting	Interventions[a] and their level of patient/family engagement	Study groups (N per group; control intervention)	Patient safety outcome[b]	Longest follow-up duration	Results pertaining to patient/family engagement in patient safety	Related publication (protocol, trial registration)
			engagement and prompting for engagement		ceftriaxone, cefuroxime, clindamycin, cefprozil, azithromycin, Cephalexin		OR 1.09; 95%CI [0.56,2.10] *p*=0.81<br><br>aOR 0.99; 95%CI [0.52,1.89] *p*=0.98 (adjusted for intervention arm, clinician type, practice type, clinician years of experience)	http://dx.doi.org/10.1136/bmjopen2017-020981
Jungo, K.T., et al.(2023)	See above, under "adverse drug events" outcome							
Kapoor, A., et al.(2023)	RCT	USA/ambulatory care	Oral & written patient education about medication safety; provision of an information hotline; systematic enquiry about patient concerns<br><br>*Level 2: Empowerment*	I:281<br>C:280<br>Coupons and assistance to increase the affordability of direct oral anticoagulants	Medication errors, ADEs<br><br>Medications: anticoagulants	3 months	No effect on medication errors or ADEs related to anticoagulants.<br><br>Medication errors (I: 234; C:214)<br>Preventable ADEs (I: 16; C:11)<br>Ameliorable ADEs (I: 3; C: 1)<br><br>Composite outcome:<br>Incidence Rate Ratio (IRR) = 1.11; 95%CI [0.93;1.32]<br>aIRR = 1.17; 95%CI [0.98; 1.42]	NA
Mortsiefer, A., et al.(2023)[d]	Cluster RCT	Germany/General practices	Multiple discussions with care team, patient, and family (including non-family care-givers) about patient preferences & concerns regarding deprescribing<br><br>*Level 1: Informing about engagement and prompting for engagement*	I:272<br>C:249<br>Standard care	Hospitalisations	12 months	No effect on hospitalisations<br>Intention to treat analysis of mean no. of hospitalisations (SD):<br>I: 0.86 (1.24) C:0.79 (1.04)<br>Incidence Rate Ratio (IRR) = 1.08; 95%CI [0.85;1.37] *p*=0.55<br>aIRR=1.08; 95%CI [0.84; 1.39] *p*=0.53<br>(adjusted for age, sex, n of chronic diseases, n of past hospitalisations at baseline)	Trial registration: https://drks.de/search/en/trial/DRKS00015055<br>Published protocol: Mortsiefer et al. 2020 https://dx.doi.org/10.1186/s13063-020-4182-x

Author, year	Type of record	Country/setting	Interventions[a] and their level of patient/family engagement	Study groups (N per group; control intervention)	Patient safety outcome[b]	Longest follow-up duration	Results pertaining to patient/family engagement in patient safety	Related publication (protocol, trial registration)
Muth, C., et al. (2016)	Cluster RCT (pilot study)	Germany/general practices	Computer-assisted discussion with patient on medication intake & polypharmacy evaluation/reduction<br><br>*Level 1: Informing about engagement and prompting for engagement*	I: 50<br>C:50<br>Standard care	Medication appropriateness<br><br>Assessed continuously, with the Medication Appropriateness Index as well as categorically<br><br><u>Medications</u>:<br>not specified	3 months	No impact on inappropriate prescription nor medication appropriateness at 3 months<br><br>Inappropriate prescriptions:<br>I: 107/393; C: 99/371<br><br>Changes of MAI score (only per protocol statistics reported):<br>I (n=46): M(SD) 0.7 (5.45)<br>C (n=47): M(SD) -0.2 (5.17)<br><br>Intercluster correlation: 0.030<br>Intercluster correlation adjusted for cluster effects: 0.039 *p*=0.460	Trial registration: https://www.isrctn.com/pdf/99691973
Muth, C., et al. (2018)	Cluster RCT	Germany/general practices	Computer-assisted discussion with patient on medication intake & polypharmacy evaluation/reduction<br><br>*Level 1: Informing about engagement and prompting for engagement*	I: 252<br>C:253<br>Standard care	Medication appropriateness<br><br>Assessed continuously, with the Medication Appropriateness Index as well as categorically<br><br><u>Medications</u>:<br>not specified	9 months	No impact on medication appropriateness<br><br>MAI score at 9 months (only per protocol statistics reported):<br>I: M(SD) 4.8 (5.2) n=238;<br>C: M(SD) 3.9 (4.9) n=228;<br>Mean difference =<br>0.6; 95%CI [-0.5 ;1.7]<br><br>Intercluster correlation: 0.0<br>Intercluster correlation adjusted for cluster effects: 0.0 *p*=0.272	Trial registrations:<br>https://clinicaltrials.gov/study/NCT01171339<br><br>https://www.isrctn.com/ISRCTN99526053
Syafhan, N. F., et al. (2021)	RCT	UK/general practices	Polypharmacy patients involved in discussion with pharmacist about medication appropriateness and management<br><br>*Level 1: Informing about engagement and prompting for engagement*	I: 181<br>C: 175<br>Standard care	Medication appropriateness assessed continuously, with the *Medication Appropriateness Index*<br><br><u>Medications</u>: consisted of a list of 102 classes of medications	6 months	No increase of improvement in MAI scores at 6 months<br><br>Mean difference in MAI scores (only per protocol statistics reported):<br>I (n=63): M(SD) 2.4 (4.8);<br>C(n=60): M(SD) 0.0 (4.0); *p*=0.879	Trial registration:<br>https://clinicaltrials.gov/study/NCT03241498

Author, year	Type of record	Country/setting	Interventions[a] and their level of patient/family engagement	Study groups (N per group; control intervention)	Patient safety outcome[b]	Longest follow-up duration	Results pertaining to patient/family engagement in patient safety	Related publication (protocol, trial registration)
Outcome: other outcomes								
Fiol-deRoque, M.A., et al. (2024)	RCT Protocol	Spain/primary healthcare centre	Patient-reported patient safety used for safety planning at the care centre's level<br><br>*Level 3: Partnership*	I: 5,900<br>C:5,900<br>Standard care	Avoidable hospitalisations; patient experiences of harm; centre's safety culture; number of actions aimed at improving patient safety in the centre	12 months	NA	Trial registration: https://clinicaltrials.gov/study/NCT05958108
Fried, T. R., et al. (2017)	RCT	USA/primary care clinics	Education & activation of older patients about value of deprescribing<br><br>*Level 2: Empowerment*	I: 64<br>C: 64<br>Standard care	Patients with medication discrepancy correction<br><br>Medications: not specified	3 months	No effect on medication discrepancy correction<br><br>No. of patients with corrected medication discrepancies:<br>I: 19/64; C: 14/64; p=0.42<br>(chi-square statistics not reported)	Trial registration: https://clinicaltrials.gov/study/NCT02501967
Serrano-Ripoll, M. J., et al. (2019)	Cluster RCT protocol	Spain/primary care centres	Patients asked to give feedback about experiences and outcomes related to patient safety<br><br>*Level 1: Informing about engagement and prompting for engagement*	I: 624<br>C: 624<br>Control group will receive the feedback reports at the end of the study	Avoidable hospitalisations	12 months	NA	Trial registration: https://clinicaltrials.gov/study/NCT03837912

Legend. RCT: randomized controlled trial; ADE: adverse drug events; GP: General Practitioner; MAI: medication appropriateness index; I: intervention; C: control

[a] Only patients and/or family engagement were extracted from broader interventions, as most interventions also involved general practitioners, pharmacists or other healthcare professionals.

[b] Only patient safety results were extracted from all reported outcomes.

[c] Falls were chosen as the outcome to include in the ADE meta-analysis.

[d] Studies with a family engagement component: interventions involving parent-child dyads or older patients with family members/non-family carers.
